# Reply to Sopek Merkaš, I.; Lakušić, N. Comment on “von Känel et al. Early Trauma-Focused Counseling for the Prevention of Acute Coronary Syndrome-Induced Posttraumatic Stress: Social and Health Care Resources Matter. *J. Clin. Med.* 2022, *11*, 1993”

**DOI:** 10.3390/jcm11226633

**Published:** 2022-11-09

**Authors:** Rebecca E. Meister-Langraf, Mary Princip, Jürgen Barth, Ulrich Schnyder, Hansjörg Znoj, Jean-Paul Schmid, Roland von Känel

**Affiliations:** 1 Department of Consultation-Liaison Psychiatry and Psychosomatic Medicine, University Hospital Zurich, University of Zurich, 8091 Zurich, Switzerland; 2 Clienia Schlössli AG, 8618 Oetwil am See, Switzerland; 3 Institute for Complementary and Integrative Medicine, University Hospital Zurich, University of Zurich, 8091 Zurich, Switzerland; 4 Medical Faculty, University of Zurich, 8006 Zurich, Switzerland; 5 Department of Health Psychology and Behavioral Medicine, University of Bern, 3012 Bern, Switzerland; 6 Department of Internal Medicine and Cardiology, Clinic Gais AG, 9056 Gais, Switzerland

We thank Merkaš and Lakušić for commenting on our recently published paper; in the paper, we suggested that resources in a patient’s social environment may moderate the benefit of one single-session trauma-focused counseling in the prevention of acute coronary syndrome (ACS)-induced posttraumatic stress disorder (PTSD) symptoms [1]. Their comment gives a comprehensive summary on the topic of ACS-induced PTSD and its treatment [2]. We agree with the authors that guidelines and standards regarding the identification and treatment of patients at high risk for developing PTSD after ACS remain lacking.

We designed the MI-SPRINT study to test whether trauma-focused psychological counseling is more effective than stress-focused counseling in preventing PTSD symptoms after acute ACS [3]. Our study showed no beneficial effect of trauma-focused counseling on PTSD symptoms; after 3 and 12 months, we found no difference in the severity of PTSD symptoms between patients with early trauma-focused counseling and those with stress-focused counseling in the total sample. However, our results suggested that psychological counseling in general might help distressed patients to prevent posttraumatic psychological responses compared with no intervention [4,5].

Importantly, PTSD symptoms that had developed after 3 months were shown to have been persistent up to 12 months after ACS, despite the delivery of one session of early psychological counseling [6]. Furthermore, as alluded to above, we showed that social support and cardiac rehabilitation act as moderators of the intervention; specifically, trauma-focused counseling was associated with fewer PTSD symptoms compared with stress-focused counseling in patients with high social support and with longer participation in cardiac rehabilitation [1]. Moreover, the data of MI-SPRINT showed that several factors contribute to identifying patients at risk for ACS-induced PTSD symptoms, such as high perceived distress during ACS [6], perception of higher harmful consequences of the illness [7], perception of a hectic hospital environment [8], sleep problems [9], and low trait resilience [10]. Screening for risk factors or specific symptoms—e.g., in the cardiac rehabilitation setting, as indicated by the Merkaš and Lakušić [1]—is important. However, we believe that screening alone may have little clinical benefit. It will be much more crucial to offer effective treatment to patients identified at high risk of developing PTSD or patients with established PTSD symptoms. In summary, further studies are needed to develop a standardized approach for the screening of patients at risk of clinically relevant, ACS-induced PTSD symptoms and to establish efficacious interventions that can be applied in a clinical setting. For instance, multisession early counseling could be elaborated and tested based on our findings to prevent the development of PTSD symptoms in patients at risk.
